# A conserved *vdcBCD* cluster encoding a vanillate/*p*-hydroxybenzoate decarboxylase catalyzes the formation of phenol from *p*-hydroxybenzoate in the cork-associated *Streptomyces graminifolii* B37 strain

**DOI:** 10.3389/fmicb.2026.1913779

**Published:** 2026-08-17

**Authors:** Marina Ruiz-Muñoz, Rebeca Cobos, Carla Calvo-Peña, Paola Callado-Tejerina, David Caparrós-Ruiz, Jordi Roselló, Juan José R. Coque

**Affiliations:** 1 Institute of Vine and Wine, University of León, León, Spain; 2 Centre for Research in Agricultural Genomics (CRAG), Campus UAB, Cerdanyola del Vallès, Barcelona, Spain; 3 Francisco Oller S. A., Cassà de la Selva, Girona, Spain

**Keywords:** aromatic acid decarboxylation, comparative genomics, cork taint, heterologous expression, *Streptomyces*, vanillate decarboxylase, yellow stain

## Abstract

Cork affected by yellow stain contains aromatic acids and volatile phenols, although the microbial reactions contributing to their formation are still poorly characterized. *Streptomyces* sp. B37, isolated from yellow-stained cork, was previously shown to convert *p*-hydroxybenzoate into phenol, but both its precise taxonomic placement and the genetic basis of this bioconversion were unknown. We combined whole-genome comparison, pangenome analysis, and heterologous expression to identify and validate the aromatic acid decarboxylation system underlying this phenotype. Phylogenomic analysis assigned strain B37 to *Streptomyces graminifolii* species. Comparative genomics showed that B37 belongs to a subclade of closely related Vanillate Decarboxylase (VDC)-positive genomes with an accessory repertoire enriched in functions related to aromatic compound uptake, regulation, redox metabolism and ring-cleavage pathways. In contrast, functions associated with primary cork-polymer degradation were not overrepresented. A conserved *vdcBCD* cluster was present in the genomes of *Streptomyces* species comprising this group and embedded in a partially conserved chromosomal neighborhood containing regulatory, oxidative and transport-related genes. Heterologous expression of the B37 *vdcBCD* cluster in *Streptomyces lividans* JI66 conferred the ability to decarboxylate *p*-hydroxybenzoate into phenol and vanillic acid into guaiacol, whereas the empty-vector control *S. lividans* JI66(pIJ699) showed no detectable decarboxylase activity. These results identify the B37 *vdcBCD* cluster as a bifunctional aromatic acid decarboxylation module and link this reaction to the formation of phenolic intermediates relevant to yellow-stained cork and cork taint-associated chemistry, including the *de novo* formation of chlorophenols and chloroanisoles.

## 1 Introduction

The most important application of cork, the outer bark of *Quercus suber* L., is the manufacture of cork stoppers for wines. Cork is a chemically restrictive plant tissue dominated by suberin, lignin, polysaccharides and extractable chemical compounds. Classical chemical analyses showed that suberin and lignin are major structural fractions of cork, whereas phenolic profiling later identified low-molecular-weight phenolic compounds such as ellagic, gallic and protocatechuic acids among cork extractives (Pereira, 1988; Santos et al., 2010). This composition makes cork a selective habitat in which associated microorganisms must tolerate both hydrophobic barriers and the presence of bioactive phenols, while potentially exploiting soluble aromatic compounds released during tissue alteration or partial polymer turnover.

This ecological context is particularly relevant for cork affected by yellow stain (YS). Occasionally, cork can develop a defect, or yellowish stain, known as YS or yellow spot. YS predominantly develops on the cork bark, particularly in areas where the bark contacts the ground, and may also cause discoloration of the adjacent cork tissue. YS is a consequence of an extensive microbial degradation of cork (Ruiz-Muñoz et al., 2025) and contains high levels of 2,4,6-trichloroanisole (2,4,6-TCA). This is not a minor issue, since once 2,4,6-TCA contaminates the cork stoppers, it can migrate into the wine producing an unpleasant off-odor characterized by moldy, musty, and earthy notes. This sensory defect can mask the natural wine aromas and compromise its organoleptic properties, a defect commonly referred to as “cork taint” (Tarasov et al., 2022). The issue of 2,4,6-TCA taint poses a substantial problem for the cork and wine industries, resulting in annual losses exceeding 10 billion dollars (Taylor et al., 2000; Zhou et al., 2024).

**FIGURE 1:**
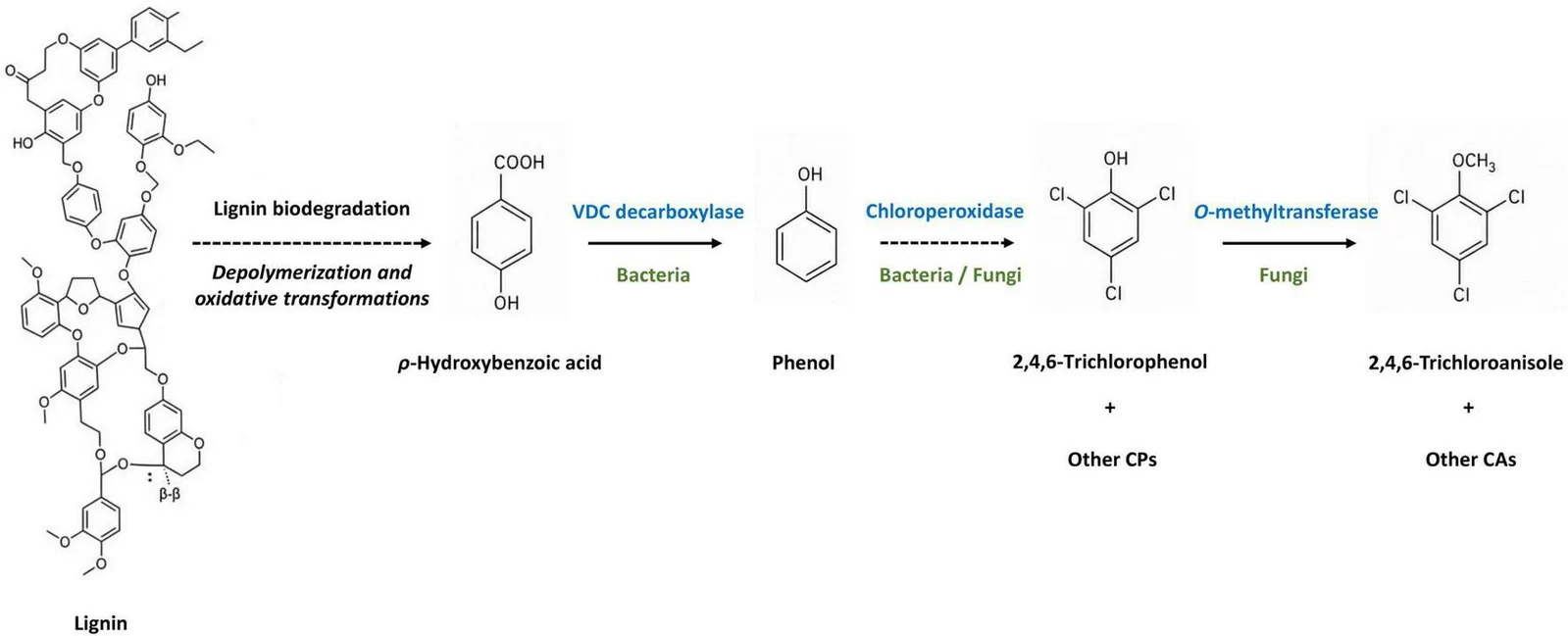
Series of events explaining “*de novo*” formation of chlorophenols (CPs) and chloroanisoles (CAs) including 2,4,6-trichloroanisole in cork affected by yellow stain. Dashed lines indicate that the process takes place in several steps. The information presented is based on Ruiz-Muñoz et al. (2025).

For a long time, the mechanisms underlying the formation and accumulation of 2,4,6-TCA in YS remained elusive. In a recent work, we demonstrated that the formation of 2,4,6-TCA is a consequence of a series of microbiologically driven biodegradation reactions involving the cork phenolic matrix. During these degradative processes, *p*-hydroxybenzoate is formed in cork and subsequently decarboxylated to phenol (Figure 1). Fungal chloroperoxidases can then chlorinate this compound to form various chlorophenols (CPs), including 2,4,6-trichlorophenol (2,4,6-TCP), the direct precursor of 2,4,6-TCA (Figure 1; Ruiz-Muñoz et al., 2025). In the same study, *Streptomyces* isolates from YS cork capable of decarboxylating *p*-hydroxybenzoate into phenol were detected. Among them, strain B37, formerly designated YS-B37, was selected for further analysis because of its cork-associated origin and its previously observed *p*-hydroxybenzoate-decarboxylating phenotype. This made B37 a suitable model to investigate the genetic basis of bacterial *p*-hydroxybenzoate decarboxylation in the context of yellow-stained cork. A previous work also showed that microorganisms isolated from cork were able to decarboxylate vanillic acid into guaiacol, linking cork-associated microbiota with the transformation of aromatic acids into volatile phenols (Álvarez-Rodríguez et al., 2003).

Members of the genus *Streptomyces* are metabolically versatile actinobacteria frequently associated with soil and plant environments (Seipke et al., 2012). Experimental studies have shown that *Streptomyces* species can transform plant-derived aromatic compounds and channel them through central aromatic-catabolic routes. Thus, *Streptomyces scabies* catabolizes ferulic and *p*-coumaric acids via the β-ketoadipate pathway, with vanillate metabolism playing a role during growth on suberin-enriched potato periderm (Khalil et al., 2019). In addition, protocatechuate catabolic genes and their regulation have been characterized in *Streptomyces*, supporting the presence of conserved routes for downstream processing of hydroxybenzoate- and vanillate-derived intermediates (Iwagami et al., 2000; Davis and Sello, 2010). However, annotated catabolic genes alone do not demonstrate activity under ecologically relevant conditions, making experimental validation necessary to connect genome content with phenotype.

Aromatic acid decarboxylation represents one transformation with direct relevance to cork-associated phenolic chemistry. In *Streptomyces* sp. D7, a non-oxidative vanillic acid decarboxylation gene cluster was shown to convert vanillic acid into guaiacol (Chow et al., 1999). Related non-oxidative hydroxyarylic acid decarboxylase systems are encoded by clustered B, C and D genes and can act on vanillate and *p*-hydroxybenzoate-like substrates (Lupa et al., 2005, 2008). Mechanistically, these systems are linked to the UbiX/UbiD family, in which a UbiX-like flavin prenyltransferase produces the prenylated FMN cofactor required by UbiD-like decarboxylases. Structural work on vanillic acid decarboxylase has further shown that VdcD acts as an allosteric activator of the UbiD-like VdcC subunit (White et al., 2015; Marshall et al., 2021).

Since the decarboxylation of *p*-hydroxybenzoate to phenol in YS-affected cork is a key step in the formation of CPs and 2,4,6-TCA in cork, this study aimed to investigate the genetic basis of this process in the cork-associated strain B37. To this end, we first sequenced the genome of strain B37 and then combined phylogenomics, comparative pangenomics and functional validation. We determined its taxonomic placement, assessed whether B37 belongs to a gene-content group carrying accessory functions associated with aromatic compound processing, and focused on the *vdcBCD* gene cluster as a candidate locus involved in *p*-hydroxybenzoate decarboxylation. By linking comparative genomics with heterologous functional validation, this study identifies a defined bacterial step in the transformation of soluble aromatic intermediates in a lignin- and suberin-rich cork environment. Beyond its ecological significance, this decarboxylation step is also of applied interest because phenol can enter the chlorophenol/chloroanisole formation route proposed for yellow-stained cork, linking aromatic acid transformation with compounds relevant to cork taint in wine (Ruiz-Muñoz et al., 2025).

## 2 Materials and methods

### 2.1 Bacterial strains, plasmids and culture conditions

*Streptomyces* sp. B37, formerly designated YS-B37, was maintained on International *Streptomyces* Project 2 medium (ISP2; Shirling and Gottlieb, 1966) agar at 28 °C. Unless otherwise stated, liquid cultures were grown in ISP2 medium at 28 °C with shaking at 180 rpm.

The plasmid vector pBluescript II KS(+) (Agilent Technologies, Santa Clara, CA, USA) was used as an intermediate cloning vector for amplification-product cloning and sequence verification. The plasmid vector pIJ699, a multi-copy positive-selection vector for *Streptomyces* (Kieser and Melton, 1988), was used for heterologous expression of the vanillate decarboxylase (*vdcBCD*) gene cluster. *Streptomyces lividans* JI66, also referred to as strain 1326 (Hopwood et al., 1983), was used as the heterologous expression host. *Escherichia coli* JM110 was used for routine cloning procedures prior to transfer into *S. lividans*.

Antibiotics were added to culture media for plasmid selection or maintenance at the following final concentrations: thiostrepton, 50 μg/mL, for selection and maintenance of recombinant *S. lividans* strains; ampicillin, 100 μg/mL, for selection of *E. coli* transformants carrying pBluescript II KS(+); kanamycin, 50 μg/mL, for selection of pIJ699-derived plasmids in *E. coli*; and streptomycin, 50 μg/mL, to maintain the streptomycin-resistant background of *E. coli* JM110 (*rpsL* mutation).

### 2.2 Genome sequencing, assembly and annotation

The genome of strain B37 was sequenced at Macrogen Inc. (Seoul, Republic of Korea). Genomic DNA was extracted following the protocol described by Hopwood et al. (1985). Purified genomic DNA was quantified using the Qubit™ 1X dsDNA HS Assay Kit (Thermo Fisher Scientific, Waltham, MA, USA). Whole-genome sequencing was performed using the PacBio Microbial Library workflow and the PacBio Revio long-read sequencing platform, with a 200 Mb sequencing output package. Raw reads were quality filtered prior to assembly. Genome assembly was performed using Canu v2.2 (Koren et al., 2017), and assembly quality was assessed using QUAST v5.3.0 (Gurevich et al., 2013) by evaluating standard metrics including genome size, N50, and GC content. Genome completeness and contamination were further assessed using CheckM2 (Chklovski et al., 2023). Genome annotation was performed using the NCBI Prokaryotic Genome Annotation Pipeline (PGAP) (Li et al., 2021).

### 2.3 Phylogenomic analysis

To determine the phylogenomic placement of strain B37 within the genus *Streptomyces*, the genome sequence was uploaded to the Type (Strain) Genome Server (TYGS)^1^ for whole-genome-based taxonomic analysis. Closely related type-strain genomes were identified by TYGS using MASH-based comparisons against the type-strain genome database (Ondov et al., 2016; Meier-Kolthoff and Göker, 2019). Pairwise intergenomic distances among the selected genomes were calculated using the Genome BLAST Distance Phylogeny (GBDP) approach with the trimming algorithm and distance formula d4, and digital DNA–DNA hybridization (dDDH) values were obtained using the recommended GGDC settings.

The whole-genome phylogenetic tree was inferred from GBDP distances using FastME v2.1.6.1 (Lefort et al., 2015), including subtree pruning and regrafting post-processing. Branch support was estimated from 100 pseudo-bootstrap replicates, and the tree was rooted at the midpoint. The genome-based tree generated by TYGS was used to assess the phylogenomic position of strain B37 and to select the closest type-strain genomes for comparative genomic analyses. Pairwise Average Nucleotide Identity (ANI) values among strain B37 and the 11 selected type-strain genomes were calculated using JSpeciesWS (Richter et al., 2016) using the ANIb algorithm (BLAST-based), which is recommended for intrageneric comparisons. Because reciprocal ANI values may differ slightly depending on query/reference direction, pairwise values were averaged to generate a symmetric ANI matrix for heatmap visualization.

### 2.4 Pangenome construction and gene-content analysis

To investigate genomic diversity and accessory functional potential within the phylogenetic lineage of strain B37, a comparative pangenome analysis was performed using the genome of B37 together with 11 closely related *Streptomyces* type-strain genomes selected based on the phylogenomic analysis described above (Supplementary Table 1; *n* = 12 genomes). This dataset was chosen to place B37 within its immediate phylogenomic context and to evaluate conservation of the *vdcBCD* cluster and associated accessory functions among its closest type-strain relatives, rather than across the whole diversity of the genus *Streptomyces*.

All genomes were annotated using Prokka v1.14.6 (Seemann, 2014), and the resulting GFF3 files were used as input for Roary v3.11.2 (Page et al., 2015). Orthologous gene clusters were inferred using a minimum BLASTp identity threshold of 85%, selected to accommodate interspecific divergence among closely related *Streptomyces* genomes while retaining functionally homologous gene clusters. The resulting files were used for downstream analyses in R v4.5.0 (R Core Team, 2025).

Gene presence–absence data were converted into a binary matrix, and pangenome compartments were defined according to the number of genomes in which each gene cluster was detected. Gene clusters present in all 12 genomes were classified as strict core genes, those present in 11 genomes as soft-core genes, those present in 3–10 genomes as shell genes, and those present in 1–2 genomes as cloud genes. This classification was used to distinguish broadly conserved genes from variable accessory genes while allowing for single-genome absences potentially associated with annotation or assembly differences.

Core-genome, pangenome, and new-gene accumulation curves were generated in R using 1,000 random genome-order permutations. For each permutation, genomes were sequentially added, and the number of gene clusters present in at least one genome, newly introduced at each addition step, and conserved across the selected genomes was calculated. For the core-genome curve, an extended core definition was applied: at each subset of *n* genomes, gene clusters present in ≥ 90% of the included genomes were counted as core. At the full set of 12 genomes, this definition corresponded to the strict core plus soft-core clusters. Mean values and standard deviations across permutations were used to visualize pangenome dynamics.

Pangenome expansion was modeled using a power-law function according to Heaps’ law, *P*(*n*) = *Kn*^γ^, where *P*(*n*) represents the number of gene clusters detected after inclusion of *n* genomes and γ indicates the degree of pangenome openness (Tettelin et al., 2005). Extended core-genome decay was fitted using an exponential decay model with an asymptote, and new-gene accumulation was fitted using a power-law decay model. Model fitting was performed using nonlinear least squares. Coefficients were estimated from the mean accumulation curves, whereas goodness-of-fit values were also calculated using all permutation points to account for the variability introduced by genome order.

To evaluate the stability of the extended core-genome decay estimate, leave-one-genome-out jackknife resampling was performed. For each of the 12 genomes removed in turn, 100 random permutations of the remaining 11 genomes were generated using the same ≥ 90% presence threshold, and the exponential decay model was refitted to each subset. The mean, standard deviation and range of the fitted plateau values were then calculated.

To explore relationships among genomes based on variable gene repertoire composition, principal component analysis and hierarchical clustering were performed using the binary gene presence–absence matrix restricted to accessory gene clusters, defined here as shell and cloud genes. Strict core and soft-core gene clusters were excluded from this analysis because they represent broadly conserved functions and contribute little to the discrimination of genome-specific or clade-associated accessory content. This accessory-focused matrix was used to evaluate whether strain B37 grouped with closely related genomes on the basis of shared variable gene content.

### 2.5 Functional annotation, enrichment analysis and curation of aromatic-compound-processing genes

Functional interpretation of the pangenome was based on Prokka annotations complemented with eggNOG-mapper functional annotations (Huerta-Cepas et al., 2017). To explore whether the VDC^+^ genomic group showed an increased representation of functions related to aromatic compound processing, accessory gene clusters were inspected and assigned to curated functional categories relevant to the ecological focus of this study. These categories included aromatic acid decarboxylation, hydroxybenzoate uptake and conversion, methoxylated aromatic compound metabolism, hydroxycinnamate-related catabolism, aromatic ring-cleavage reactions, oxidative enzymes, transport and transcriptional regulation.

To identify gene families associated with the VDC^+^ genomic group, an exploratory enrichment analysis was performed using the Roary gene presence–absence matrix. Genomes containing the *vdcBCD* cluster were designated VDC^+^; those lacking it were VDC^–^. Gene clusters present in fewer than three genomes were excluded from enrichment testing to reduce the influence of rare cloud genes and to focus on broadly distributed accessory families. For each remaining orthologous gene cluster, a one-sided Fisher’s exact test was used to assess enrichment in the VDC^+^ group relative to the remaining genomes. *P*-values were adjusted for multiple testing using the Benjamini–Hochberg false discovery rate correction (Benjamini and Hochberg, 1995). Gene families with adjusted *p*-values < 0.05 were considered significantly enriched. Curated functional categories were then compared at module level by counting the number of assigned gene clusters in each genome and normalizing this value by the total number of gene clusters detected in that genome. Mean normalized values were calculated separately for the VDC^+^ genomic group and for the remaining genomes, and the results were expressed as VDC^+^ genomic group/outgroup ratios.

### 2.6 Identification and genomic context of the *vdcBCD* aromatic acid decarboxylation locus

To identify the aromatic acid decarboxylation system selected for functional validation, the annotated genome of strain B37 was inspected for genes related to bacterial non-oxidative hydroxyarylic acid decarboxylase/phenol carboxylase systems. Particular attention was paid to UbiX-like flavin prenyltransferases, UbiD-like decarboxylases and small VdcD-like accessory proteins within the broader set of aromatic-metabolism-associated genes identified during functional curation. Through manual inspection of candidate UbiD/UbiX-associated regions, a three-gene cluster comprising *vdcB*, *vdcC* and *vdcD* was identified in strain B37. The organization of this locus was compared with previously described BCD-type aromatic acid decarboxylation systems.

To evaluate the genomic context of the *vdcBCD* locus and its relationship with neighboring aromatic-metabolism-associated genes, flanking regions were examined for regulatory, transport-related, oxidoreductase and other catabolic genes. Homologous regions in closely related genomes were compared by synteny analysis using clinker^2^ (Gilchrist and Chooi, 2021).

### 2.7 Heterologous expression of the VDC gene cluster

To functionally characterize the VDC system identified in strain B37, the complete gene cluster was expressed in *S. lividans* JI66, a genetically tractable heterologous host that showed no detectable endogenous aromatic acid decarboxylation activity under the assay conditions and whose genome lacks a complete *vdcBCD* cluster.

#### 2.7.1 Primer design and PCR amplification

Specific oligonucleotide primers for amplifying the *vdc*BCD cluster, comprising *vdcB*, *vdcC*, and *vdcD* genes, were designed using Primer3 v2.3.7 (Untergasser et al., 2012) based on the complete genome sequence of strain B37. The amplified region was designed to include the complete *vdcBCD* cluster together with the upstream promoter region of *vdcB*. The forward and reverse primers included *BamH*I restriction sites at their 5’ ends, preceded by three additional nucleotides to ensure efficient enzyme cleavage (Supplementary Table 2).

The fragment amplified for heterologous expression spanned positions 9,385,846–9,388,884, corresponding to a 3,039-bp genomic amplicon that included 507 bp upstream of the *vdcB* coding sequence and 179 bp downstream of the *vdcD* stop codon. Including the non-genomic *BamHI*-containing 5’ tails added by both primers, the expected PCR product size was 3,057 bp. Inspection of the upstream region of *vdcB* revealed promoter-like elements compatible with conserved *Streptomyces* motifs described by Lee et al. (2019), including a TGAC motif, two -10-like TANNNT-compatible motifs, and a putative Shine–Dalgarno/ribosome-binding site sequence located 9 bp upstream of the *vdcB* start codon.

PCR amplification was carried out using Platinum SuperFi II DNA Polymerase (Thermo Fisher Scientific) to minimize replication errors. The reaction mixture (50 μL) contained 1 × Platinum SuperFi II buffer, 200 μM each dNTP, 0.5 μM each primer, Platinum SuperFi II DNA Polymerase, and approximately 25 ng of genomic DNA extracted from strain B37. Thermal cycling conditions were as follows: initial denaturation at 98 °C for 2 min; 30 cycles of 98 °C for 30 s, annealing at 60 °C for 10 s, and extension at 72 °C for 2 min; followed by a final extension at 72 °C for 5 min. PCR products were purified using the FavorPrep™ GEL/PCR Purification Mini Kit (Favorgen Biotech Corp., Ping-Tung, Taiwan).

#### 2.7.2 Construction of the recombinant plasmid

The purified PCR fragment was first cloned into the *BamH*I site of the pBluescript II KS(+) vector for sequence verification. Recombinant plasmids were screened, and the accuracy of the amplified sequence was confirmed by Sanger sequencing using the primers listed in Supplementary Table 2. For cloning of the B37 *vdcBCD* cluster into *S. lividans* JI66, pIJ699 was isolated from transformed *E. coli* JM110. After plasmid purification, it was double-digested with *BamH*I and *Bgl*II restriction endonucleases. The *Bgl*II fragment containing the pIJ101 origin of replication and the thiostrepton resistance gene was purified from agarose gels. The *BamH*I-digested *vdcBCD* insert was then ligated into the *Bgl*II-linearized pIJ699 vector, generating compatible cohesive-end junctions. Recombinant plasmids carrying the insert of the expected size were selected for transformation into *S. lividans*.

#### 2.7.3 Preparation of *Streptomyces* protoplasts

Protoplasts of *S. lividans* JI66 were prepared following a modified version of the protocol described by Kieser et al. (2000). Briefly, spores were inoculated into yeast extract–malt extract (YEME) medium supplemented with 5 mM MgCl_2_⋅6H_2_O and 0.5% glycine and incubated at 30 °C with shaking (200 rpm) for 36–40 h. Lysozyme was added to a final concentration of 1 mg/mL, and the suspension was incubated at 30 °C for 30–45 min with gentle mixing to generate protoplasts. Lysozyme treatment was monitored by optical microscopy to assess protoplast formation. The protoplast suspension was filtered through sterile cotton wool, centrifuged (1000 × *g*, 7 min), and gently resuspended in P buffer to obtain a concentrated suspension.

#### 2.7.4 Transformation of *S. lividans* protoplasts

Transformation was performed using freshly prepared protoplasts. Briefly, protoplasts were mixed with 2–5 μg of recombinant plasmid DNA (or empty pIJ699 as a control). Immediately, 0.5 mL of 25% (w/v) PEG 1000 in P buffer was added, and the mixture was gently mixed. After a short incubation (< 3 min), 5 mL of P buffer was added. The mixture was centrifuged (1000 x *g*, 7 min), and protoplasts were gently resuspended and plated onto R5 agar plates without antibiotics. Plates were incubated at 30 °C for 14–20 h to allow regeneration. Selection was performed by overlaying the plates with thiostrepton dissolved in DMSO to achieve a final concentration of approximately 50 μg/mL in the plate. Plates were further incubated at 30 °C for 5–7 days until colonies appeared. Individual transformants were picked on R5 agar plates supplemented with thiostrepton (50 μg/mL). The presence of the *vdcBCD* cluster was confirmed by colony PCR using the primers described in section “2.7.1 Primer design and PCR amplification**”**. Plasmid DNA from selected transformants was subsequently extracted and verified by diagnostic restriction digestion.

### 2.8 Aromatic acid decarboxylation assays

To evaluate the activity of the VDC system, resting-cell assays were performed with recombinant strains expressing the gene cluster. The decarboxylation of *p*-hydroxybenzoate and vanillic acid was assessed by monitoring the formation of phenol and guaiacol, respectively.

Control experiments included: (i) *S. lividans* carrying the empty vector, *S. lividans* JI66(pIJ699), as a negative control, (ii) *Streptomyces* sp. B37 as a positive control, (iii) resting-cell suspensions incubated in phosphate buffer without chemical substrates to assess background activity, and (iv) non-inoculated controls containing phosphate buffer and each substrate, to assess abiotic substrate stability and exclude non-biological formation of phenol or guaiacol.

Strains were grown in 500 mL Erlenmeyer flasks containing 100 mL of ISP2 medium at 28 °C and 200 rpm for 72 h. Cultures were inoculated with ten agar plugs (9 mm diameter) excised from 7-day-old ISP2 agar cultures of each strain. For recombinant *S. lividans* strains, thiostrepton (50 μg/mL) was added to the culture media. Cells were harvested by centrifugation under sterile conditions and washed twice at room temperature with 40 mL of 0.9% NaCl. The washed biomass was resuspended in 25 mM phosphate buffer (pH 7.0) and adjusted to an equivalent OD_600_ of 3.0, measured after appropriate dilution and homogenization of the cell suspension. Resting-cell suspensions were then supplemented with 12 mM *p*-hydroxybenzoate or vanillic acid (Thermo Fisher Scientific) in a final volume of 20 mL. For recombinant *S. lividans* strains, thiostrepton (50 μg/mL) was also added. Resting-cell suspensions were incubated at 30 °C with agitation (200 rpm) in an orbital shaker. At defined time points (0 h, 20 min, 40 min, 1 h, 2 h, 4 h, 8 h, and 24 h), 1 mL aliquots were withdrawn for analysis. Reactions were stopped by adding 1 mL of methanol and 10 μL of formic acid to acidify the samples. After centrifugation (7800 x *g*, 30 min, 4 °C), the supernatants were collected for analysis by high-performance liquid chromatography (HPLC).

Samples were filtered through 0.45 μm pore-size Corning^®^ Costar^®^ Spin-X^®^ centrifuge tube filters (Merck KGaA, Darmstadt, Germany) and stored at −20 °C until analysis. Aliquots (10 μL) were injected and separated on a LiChrospher RP18 analytical column (4.6 × 250 mm, 5 μm) (Teknokroma, San Cugat del Vallés, Spain) using an acetonitrile–water–formic acid mobile phase (20:80:1, v/v/v) at a flow rate of 1 mL/min. Compounds were monitored at 275 nm using an Agilent 1200 Series HPLC system (Agilent Technologies) equipped with a quaternary pump (G1311A), autosampler (G1329A), diode array detector (G7115A), and autosampler thermostat (G1330B). Phenol and guaiacol (Acros Organics, Geel, Belgium) were identified based on retention time and UV–Vis spectra compared to commercial standards. Quantification was performed using calibration curves generated from standard solutions of *p*-hydroxybenzoate, vanillic acid, phenol, and guaiacol prepared in methanol over an appropriate concentration range. All experiments were performed in three independent biological replicates, each with two technical replicates.

### 2.9 Statistical analysis

Technical replicates were averaged prior to statistical analysis, and biological replicates were used for inference. All analyses were performed on log10(x + 1)-transformed concentrations to improve model fit and variance structure, particularly because product concentrations included zero or near-zero values. Time-course data for *p*-hydroxybenzoate, phenol, vanillic acid, and guaiacol were analyzed using linear mixed-effects models with strain, time, and their interaction as fixed effects, and biological replicate nested within strain as a random intercept using the lme4 (Bates et al., 2015) and lmerTest (Kuznetsova et al., 2017) packages in R. Time was treated as a categorical factor to allow comparison among strains at each sampling point without assuming a linear temporal response. For vanillic acid, the estimated variance of the random effect was zero; therefore, the mixed model was equivalent to the corresponding two-way ANOVA, although the same model formulation was retained for consistency across compounds.

Model assumptions were evaluated by inspection of residual plots and Levene’s test (Levene, 1960). Significance of fixed effects was assessed using Type III analysis of variance with Satterthwaite’s approximation. *Post-hoc* pairwise comparisons among the three active strains (B37 and the two VDC transformants) were performed with Tukey adjustment. Comparisons of each active strain against *S. lividans* JI66(pIJ699) at each time point were performed with Sidak adjustment. Partial eta squared (partial η^2^) was calculated from the corresponding fixed-effect linear model on the transformed scale. Statistical analyses were performed in R, and differences were considered significant at *p* < 0.05.

## 3 Results

### 3.1 Genome features and phylogenomic placement of strain B37

**FIGURE 2:**
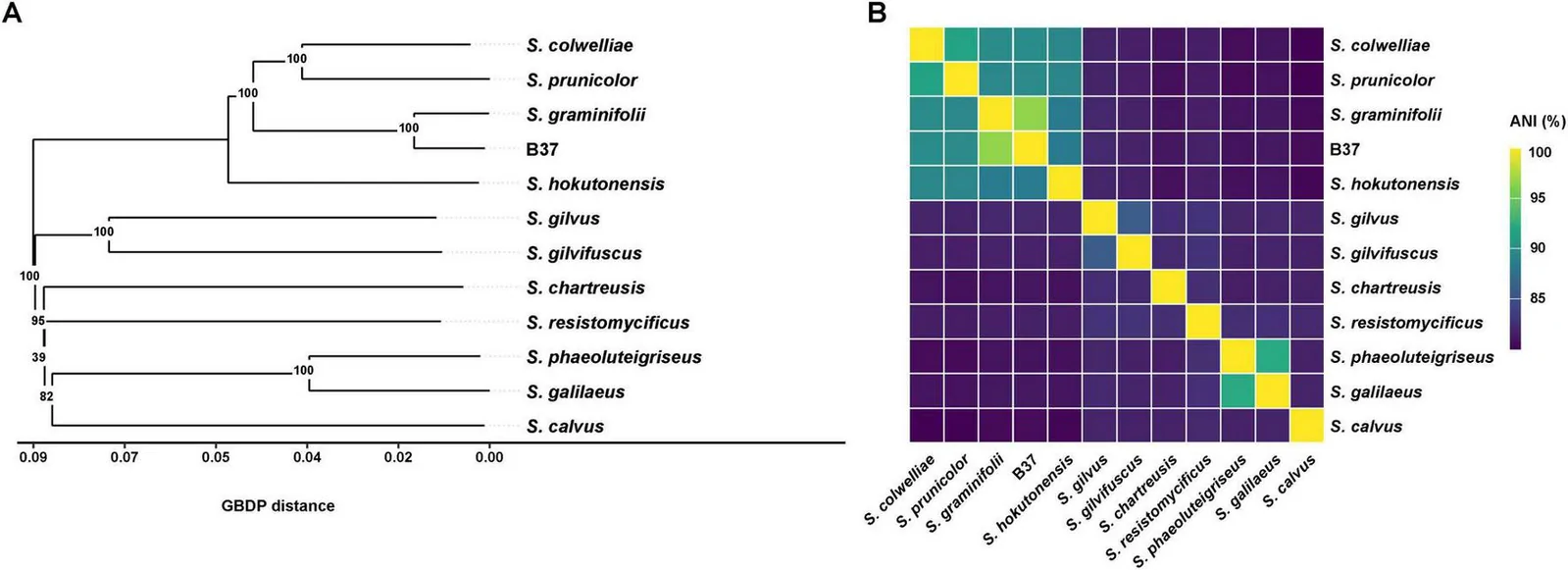
Phylogenomic placement and pairwise genomic relatedness of strain B37. **(A)** Whole-genome-based phylogenetic tree showing the position of strain B37 among closely related *Streptomyces* type-strain genomes. The tree was inferred with FastME from GBDP distances calculated from genome sequences (100 pseudo-bootstrap replicates; midpoint rooting). **(B)** Pairwise ANI heatmap among strain B37 and the same 11 type-strain genomes selected for comparative pangenome analysis (Supplementary Table 1).

The genome of strain B37 was sequenced using PacBio long-read technology and assembled into a single linear contig, with a total genome size of 10,009,576 bp and a GC content of 70.6%. According to the NCBI PGAP annotation, the genome contains 8,802 coding sequences (CDSs), along with 18 rRNA genes (six 5S, six 16S and six 23S rRNA genes), 72 tRNA genes, and 10 ncRNA genes. Genome quality assessment using CheckM2 indicated a high-quality assembly, with an estimated completeness of 99.91% and low contamination (0.51%). To determine its taxonomic position, a phylogenomic analysis was performed using the TYGS platform, including closely related type strains (Supplementary Table 1). The genome-based phylogeny placed strain B37 within a well-supported clade together with *Streptomyces graminifolii* NBRC 109806 (Figure 2A). This assignment was further supported by a dDDH value of 74.8%, exceeding the accepted species delineation threshold. Consistent with this result, pairwise ANI analysis showed that B37 shared its highest genomic similarity with *S. graminifolii* NBRC 109806, with an average reciprocal ANI value of 96.6%, confirming its classification within this species (Figure 2B). In contrast, ANI values with the remaining type strains were substantially lower (< 92%).

### 3.2 Pangenome structure and accessory gene-content relationships among strain B37 and closely related *streptomyces* genomes

Pangenome analysis was performed using strain B37 and 11 closely related *Streptomyces* type-strain genomes selected from the phylogenomic analysis (total *n* = 12; Supplementary Table 1). Orthologous clustering identified a total of 46,642 gene clusters across the analyzed genomes. The strict core genome, defined as gene clusters present in all 12 genomes, comprised 1,965 clusters, representing only 4.21% of the total pangenome. An additional 413 gene clusters were detected in 11 of the 12 genomes and were classified as soft-core genes. Together, strict and soft-core genes accounted for 2,378 clusters, corresponding to 5.10% of the pangenome.

Most of the pangenome corresponded to accessory genes: 6,225 clusters were classified as shell genes (present in 3–10 genomes) and 38,039 as cloud genes (present in only one or two genomes), representing 13.35% and 81.56% of the pangenome, respectively. This distribution indicates an accessory-rich pangenome, with a small conserved genomic backbone and a large fraction of rare or strain-restricted gene clusters.

**FIGURE 3:**
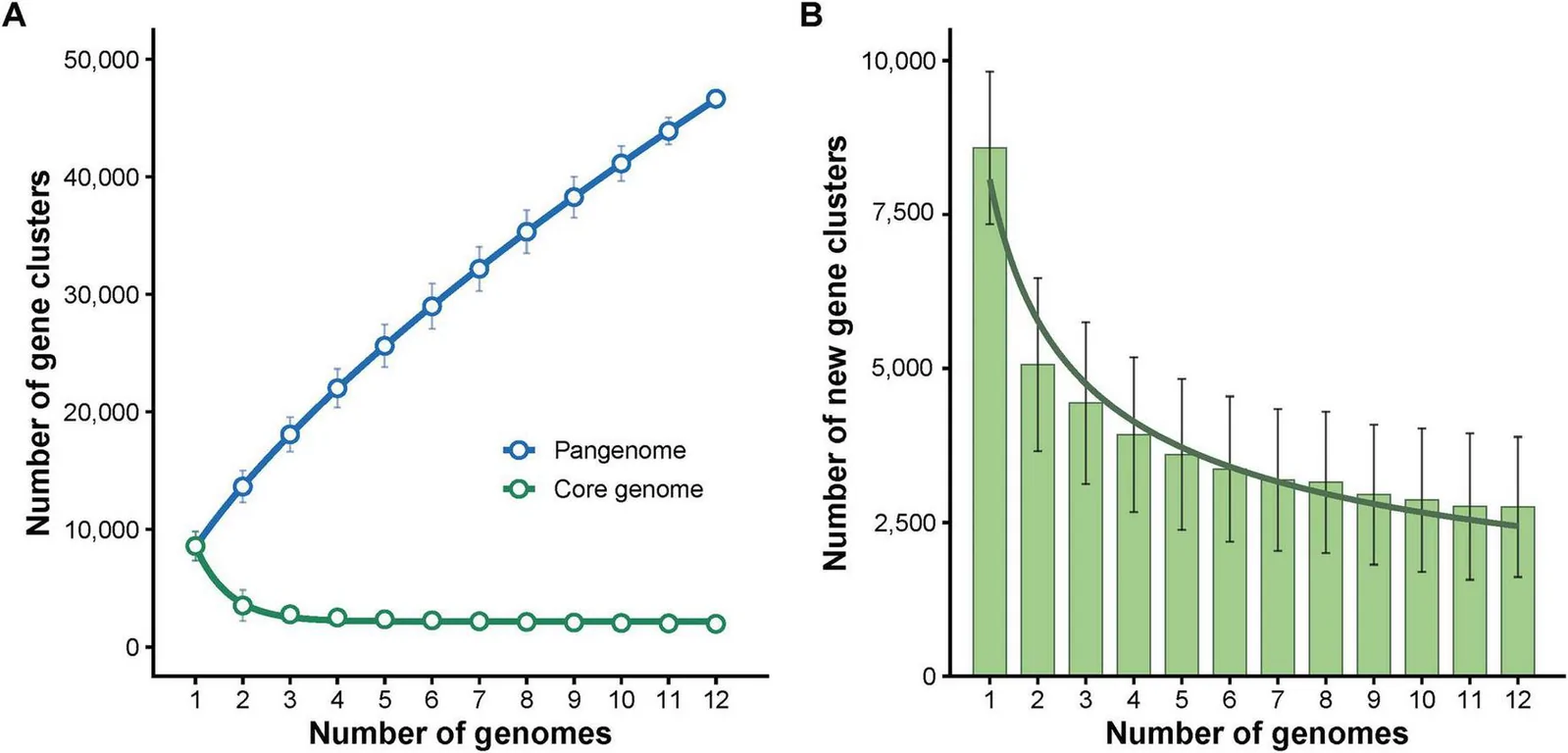
Pangenome dynamics of strain B37 and closely related *Streptomyces* type-strain genomes. **(A)** Pangenome and extended-core genome accumulation curves based on 1,000 random genome-order permutations. The extended core was defined using a ≥ 90% presence threshold for each genome subset. **(B)** New-gene accumulation curve showing the number of previously undetected gene clusters contributed by each additional genome. Values represent means across permutations; error bars indicate standard deviation.

Permutation-based pangenome curves further supported this pattern (Figure 3). The pangenome increased continuously as additional genomes were incorporated and showed no evidence of saturation within the analyzed dataset. Fitting the pangenome accumulation curve to a power-law model yielded the equation *P*(*n*) = 8521⋅*n*^0.684^, with a Heaps’ law exponent of γ = 0.684, indicating a substantially open pangenome.

An extended core definition was used for the core-genome accumulation curve, considering gene clusters present in ≥ 90% of the genomes in each subset. At the full set of 12 genomes, this extended core comprised the strict plus soft-core clusters, i.e., 2,378 gene clusters. The curve decreased rapidly as genome number increased and approached an asymptote (Figure 3A). Fitting an exponential decay model gave an estimated asymptote of approximately 2,165 gene clusters (*C*(*n*)= 26843.2⋅*e*^−1.4343*n*^ +  2165; R^2^ = 0.9925), suggesting that the extended-core curve tends toward stabilization within the analyzed dataset. The new-gene curve decreased with additional genomes but remained high even at the twelfth genome (Figure 3B), indicating that each newly added genome still contributed a considerable number of previously undetected clusters.

Leave-one-genome-out jackknife resampling, with 100 random genome-order permutations per reduced dataset, yielded a mean extended-core asymptote of 2,190 ± 54 gene clusters, with values ranging from 2,155 to 2,353. This result is consistent with the observed extended core at *n* = 12 (2,378) and supports the stability of the fitted extended-core decay pattern.

**FIGURE 4:**
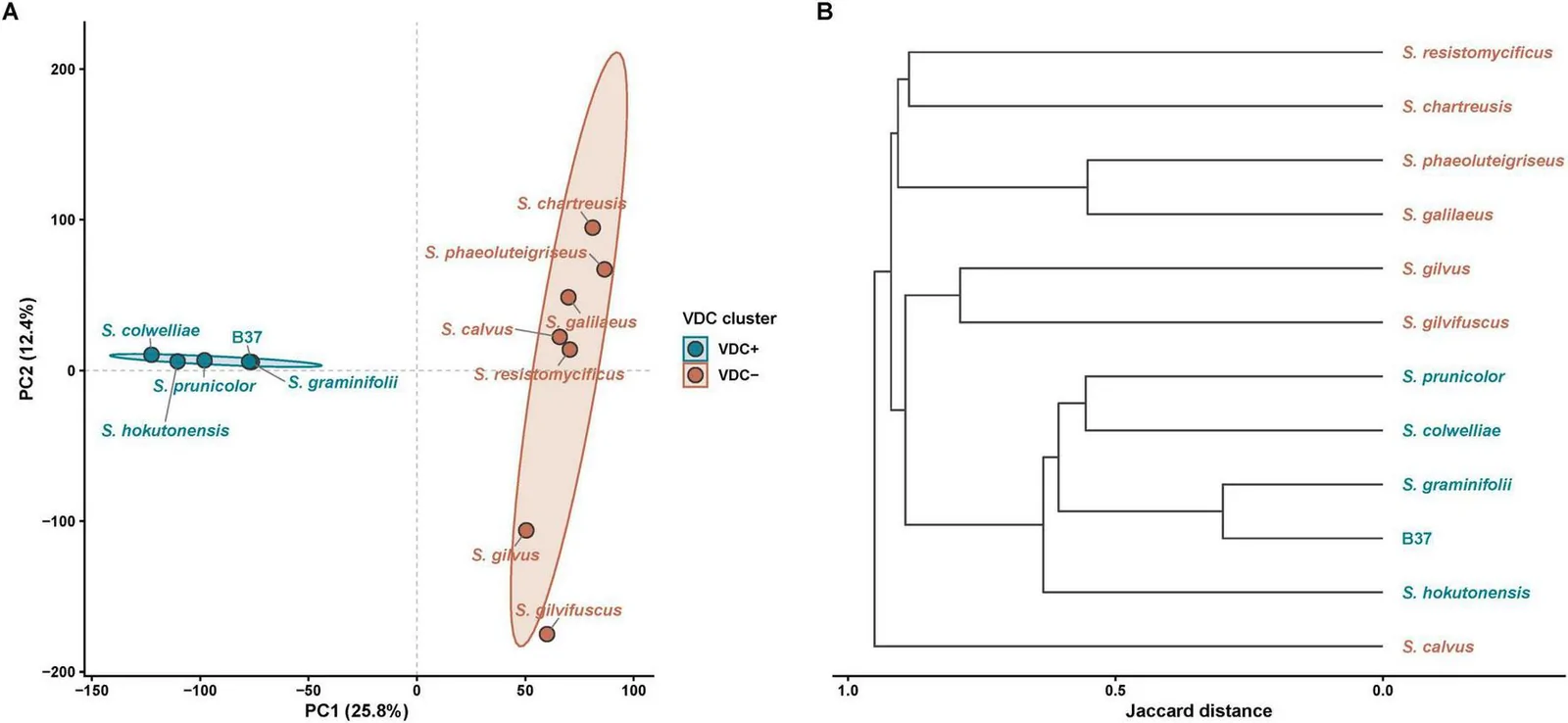
Gene-content relationships among strain B37 and closely related *Streptomyces* genomes. **(A)** Principal component analysis based on the accessory-gene presence–absence matrix, including shell and cloud gene clusters. **(B)** Hierarchical clustering based on the same presence–absence profiles.

To evaluate whether gene-content patterns reflected the phylogenomic placement of B37, principal component analysis was performed on the accessory-gene presence–absence matrix, including shell and cloud gene clusters (Figure 4A). The first two principal components explained 25.8% and 12.4% of the total variance, respectively, together accounting for 38.2% of the gene-repertoire variation among the analyzed genomes. B37 was positioned almost overlapping with *S. graminifolii* in the PC1–PC2 space, showing the shortest distance to this species (Euclidean distance = 0.204), followed by *S. hokutonensis* (2.05), *S. prunicolor* (3.06), and *S. colwelliae* (7.62). In contrast, all remaining genomes were clearly separated from B37 in the PCA space, with distances greater than 66, indicating a markedly distinct gene-content composition.

Hierarchical clustering based on the same accessory-gene presence–absence matrix provided a complementary view of these relationships (Figure 4B). Consistent with the PCA, B37 appeared adjacent to *S. graminifolii* and within the same accessory gene-content group as *S. hokutonensis*, *S. colwelliae*, and *S. prunicolor*. This pattern agrees with the phylogenomic placement of B37 and indicates that these genomes share a more similar gene-repertoire composition compared with the remaining analyzed *Streptomyces* species.

### 3.3 Comparative accessory-gene profiling suggests enrichment of aromatic-compound-processing traits in the VDC^+^ genomic group

Because the strict core was small and the cloud genome was highly variable, functional analyses focused on accessory gene clusters shared by B37 and its closest gene-content relatives. This made it possible to examine whether the VDC^+^ genomic group carried accessory functions potentially associated with the processing of plant-derived aromatic compounds. To identify gene families associated with the VDC^+^ genomic group, a one-sided Fisher’s exact test on the Roary gene presence–absence matrix was performed, followed by Benjamini–Hochberg correction. This analysis identified 100 orthologous gene families showing nominal enrichment in the VDC^+^ genomic group (*p* < 0.05), including 44 families that remained significant after Benjamini–Hochberg correction and were exclusively detected in all VDC^+^ genomes and absent from the outgroup (Supplementary Table 3).

Functional inspection of the enriched families revealed that the VDC^+^ genomic group was defined not only by the *vdcBCD* decarboxylation module, but also by a broader accessory repertoire related to the handling of aromatic compounds.

Several enriched families were associated with hydroxybenzoate uptake and conversion, including *praI*-like 4-hydroxybenzoate monooxygenases and the aromatic acid transporter *pcaK*. Additional families were linked to hydroxycinnamate-related metabolism, including *hcaB*, *hcaR*, *mhpA*, *mhpB* and *fldA*-like functions, suggesting an increased representation of genes involved in transformations of phenylpropanoid-derived compounds. The VDC^+^ genomic group also carried enriched families associated with downstream aromatic-ring processing, including hydroxyquinol 1,2-dioxygenases such as *npcC* and *chqB*. Redox-associated functions were also represented, including quinone oxidoreductases, glutathionyl-hydroquinone reductase-like genes, multicopper oxidase and peroxidase-like enzymes.

**FIGURE 5:**
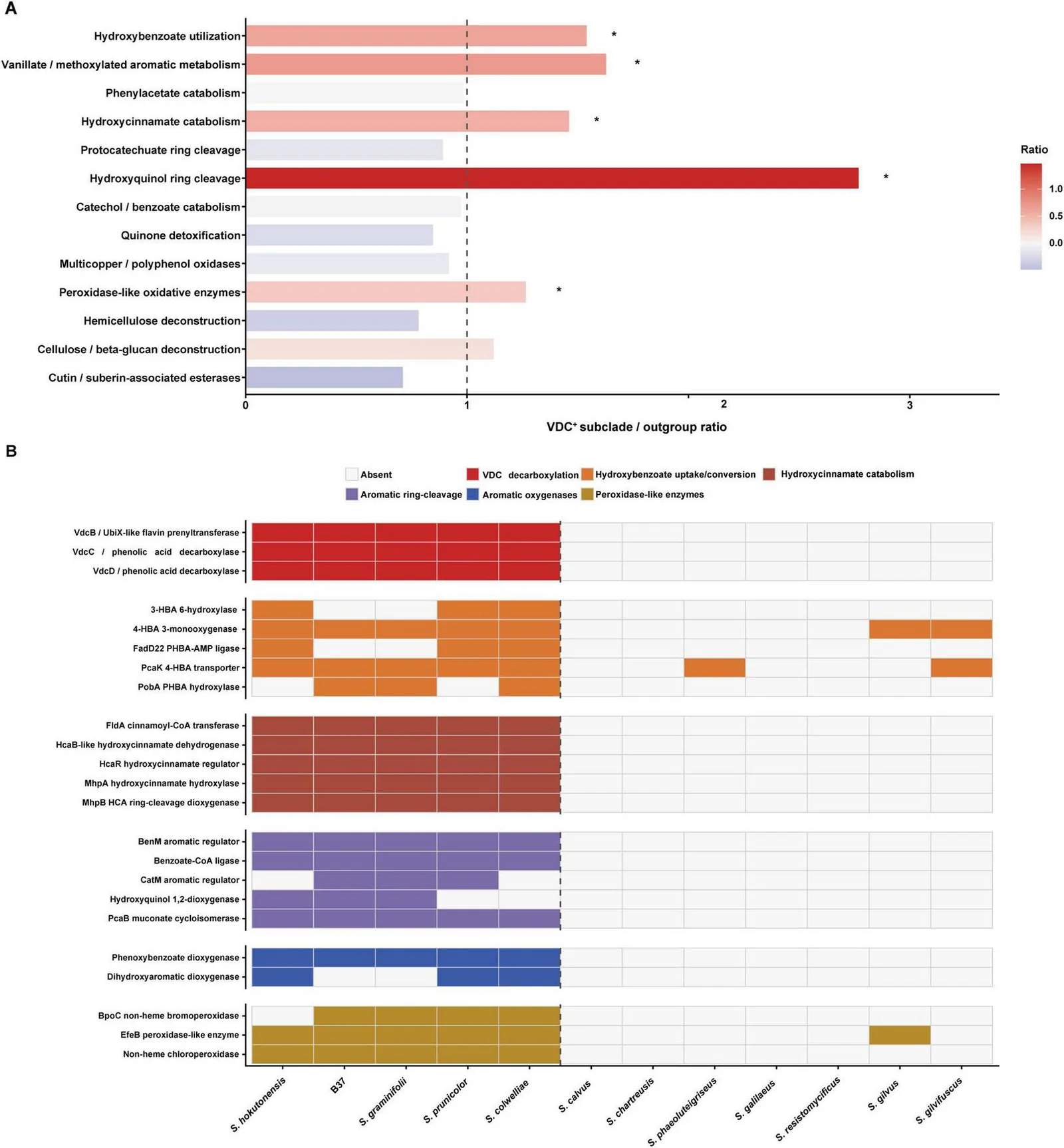
Enrichment and comparative profiling of aromatic-compound-associated functions in the VDC^+^ genomic group. **(A)** Relative representation of curated functional modules in the VDC^+^ genomic group compared with outgroup genomes. Bars represent the ratio of mean normalized gene cluster counts between the VDC^+^ genomic group and the outgroup. The dashed vertical line indicates a ratio of 1. Asterisks (*) indicate modules significantly enriched in the VDC^+^ genomic group (one-sided Fisher’s exact test with Benjamini–Hochberg correction; adjusted *p*-value < 0.05). **(B)** Presence/absence profiles of selected significantly enriched or functionally relevant genes. The dashed vertical line separates VDC^+^ genomes from outgroup genomes.

At the broader functional-category level, the strongest relative signal corresponded to hydroxyquinol ring cleavage, followed by categories related to methoxylated aromatic metabolism, hydroxybenzoate utilization and hydroxycinnamate catabolism (Figure 5A). Peroxidase-like oxidative enzymes also showed a moderate increase in the VDC^+^ genomic group. In contrast, broader categories related to primary plant-polymer degradation, including hemicellulose deconstruction and cutin/suberin-associated esterases, were not overrepresented in the VDC^+^ genomic group.

The distribution of selected representative genes across individual genomes further supported this functional pattern (Figure 5B). The complete *vdcBCD* locus was conserved in all VDC^+^ genomes and absent from the outgroup. This same group also showed preferential conservation of representative genes involved in hydroxybenzoate conversion, hydroxycinnamate metabolism, hydroxyquinol ring cleavage, quinone reduction and peroxidase-like activity, whereas these functions were absent or more sporadically distributed among the outgroup genomes.

This comparative accessory-gene profile does not support broad primary degradation of cork polymers by B37 and its closest relatives. Instead, the enriched repertoire points mainly to uptake, redox balancing and transformation of soluble aromatic intermediates that may arise during cork alteration. The conserved *vdcBCD* cluster was therefore examined in detail as an experimentally testable component of this aromatic-compound-processing repertoire.

### 3.4 Conservation and genomic context of the *vdcBCD* aromatic acid decarboxylation module

The organization of the B37 cluster region matched the canonical structure described for bacterial non-oxidative hydroxyarylic acid decarboxylase/phenol carboxylase systems. The cluster comprised a gene encoding a UbiX-like flavin prenyltransferase (VdcB; 198 amino acids), a larger UbiD-like phenolic acid decarboxylase component (VdcC; 474 amino acids), and a small accessory protein (VdcD; 74 amino acids). In the B37 strain, the three coding sequences were arranged in the order *vdcB–vdcC–vdcD* and separated by short intergenic regions of 69 bp between *vdcB* and *vdcC*, and 34 bp between *vdcC* and *vdcD*. The complete module spanned approximately 2.35 kb.

This compact organization was conserved across the VDC^+^ genomes analyzed. In *S. graminifolii*, *S. prunicolor*, *S. hokutonensis* and *S. colwelliae*, the three genes were retained in the same transcriptional order, *vdcB–vdcC–vdcD*, with similarly short intergenic regions. VdcB and VdcC showed high sequence similarity across the five genomes, with amino acid identities ranging from 96.46 to 97.98% and from 96.20 to 97.26%, respectively. VdcD was also retained in all genomes carrying the complete module, although it showed greater variation in sequence conservation and predicted length, with amino acid identities ranging from 87.67 to 91.89% (Table 1).

**TABLE 1:** Amino acid identity of B37 Vdc proteins against homologues detected in closely related *Streptomyces* type-strain genomes.

Protein	*S. graminifolii*	*S. prunicolor*	*S. hokutonensis*	*S. colwelliae*
VdcB	193/198 (97.47%)	194/198 (97.98%)	191/198 (96.46%)	193/198 (97.47%)
VdcC	461/474 (97.26%)	457/474 (96.41%)	456/474 (96.20%)	461/474 (97.26%)
VdcD	66/74 (89.19%)	68/74 (91.89%)	64/73 (87.67%)	66/74 (89.19%)

Values indicate identical amino acids over aligned protein length, followed by percentage identity. B37 proteins were used as query sequences.

**FIGURE 6:**
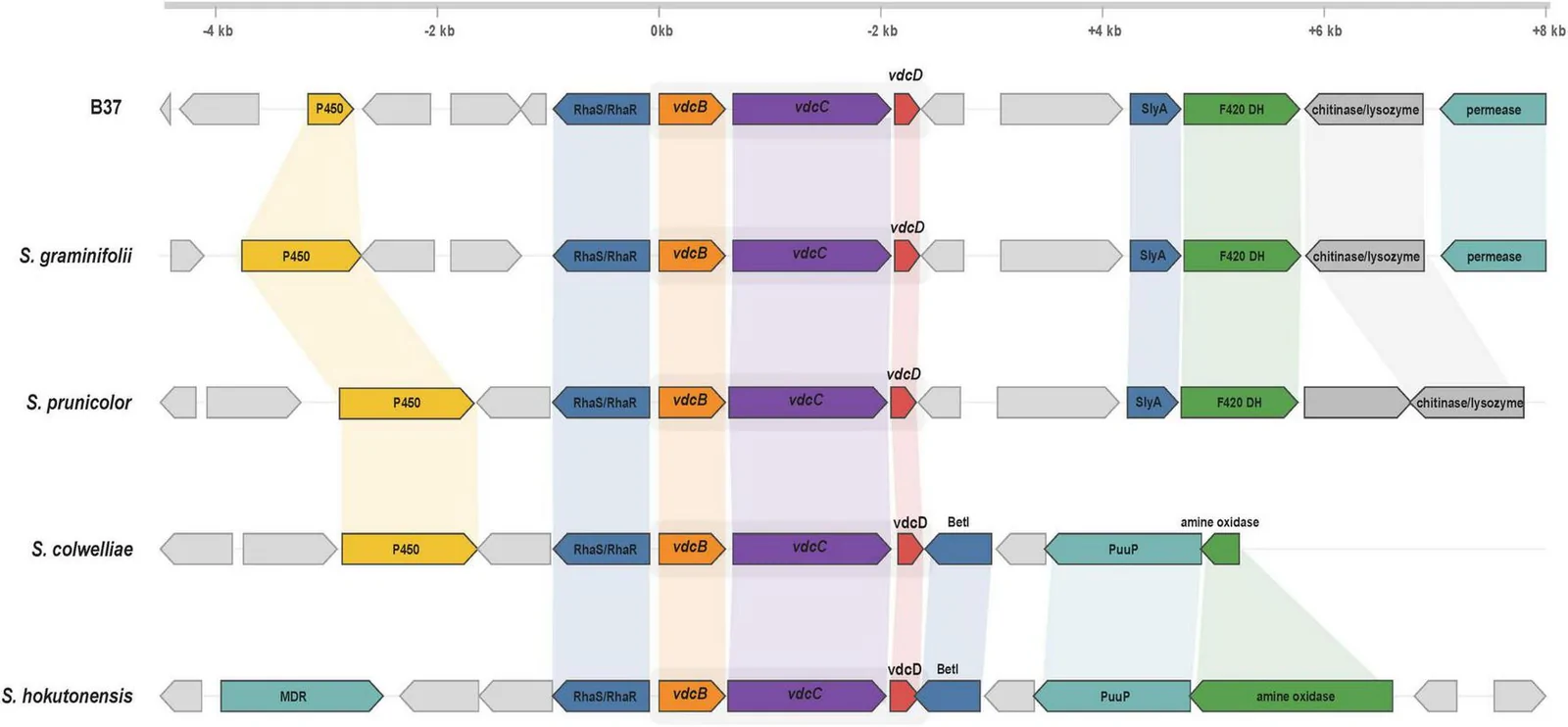
Genomic context of the *vdcBCD* aromatic acid decarboxylation module in strain B37 and closely related VDC^+^ *Streptomyces* genomes. Arrows represent predicted coding sequences and their transcriptional orientation. Colors denote functional categories: *vdcB*, orange; *vdcC*, purple; *vdcD*, red; transcriptional regulators, blue; P450/redox-associated enzymes, yellow/green; transport-related proteins, turquoise; and hypothetical proteins, gray. Homologous genes are connected by colored links, with only similarities ≥ 80% shown. Abbreviations: RhaS/RhaR, AraC-family regulator; SlyA, MarR-family regulator; P450, cytochrome P450 monooxygenase; F420 DH, F420-dependent dehydrogenase; MDR, putative multidrug resistance transporter; PuuP, putrescine importer; BetI, BetI-family regulator.

Synteny analysis showed that the *vdcBCD* module was embedded within a partially conserved local genomic context (Figure 6). Immediately upstream of the cluster, all five VDC^+^ genomes carried an AraC-family transcriptional regulator annotated as RhaS/RhaR-like, located in divergent orientation relative to *vdcB*, separated from the cluster by a short intergenic region of approximately 87–88 bp. Although the regulatory architecture of this region was not experimentally tested, this conserved head-to-head arrangement suggests that the *vdcBCD* cluster is associated with local transcriptional control. In addition, a PikC-like cytochrome P450 monooxygenase or P450-related coding sequence was detected near the *vdcBCD* module in B37, *S. graminifolii*, *S. colwelliae* and *S. prunicolor*, whereas this element was not detected in the corresponding upstream region of *S. hokutonensis*.

In contrast to the conserved central module and upstream regulatory arrangement, the downstream region was more variable among genomes. B37 and *S. graminifolii* showed a highly similar downstream organization, including hypothetical coding sequences, a MarR-family transcriptional regulator annotated as SlyA-like, an F420-dependent dehydrogenase, a putative chitinase/lysozyme, permease-associated genes and carbohydrate transport-related proteins. *S. prunicolor* retained part of this downstream architecture, including the SlyA-like regulator, F420-dependent dehydrogenase and permease-associated genes. By contrast, *S. colwelliae* and *S. hokutonensis* displayed a different downstream arrangement, including BetI-family transcriptional regulators, putrescine importer-associated proteins and amine-oxidase-like genes.

Across the five genomes, conservation was strongest for the VDC cluster and the divergent RhaS/RhaR-like regulator, whereas the downstream region varied in gene content and organization.

### 3.5 Functional validation of the *vdcBCD* cluster by heterologous expression

**FIGURE 7:**
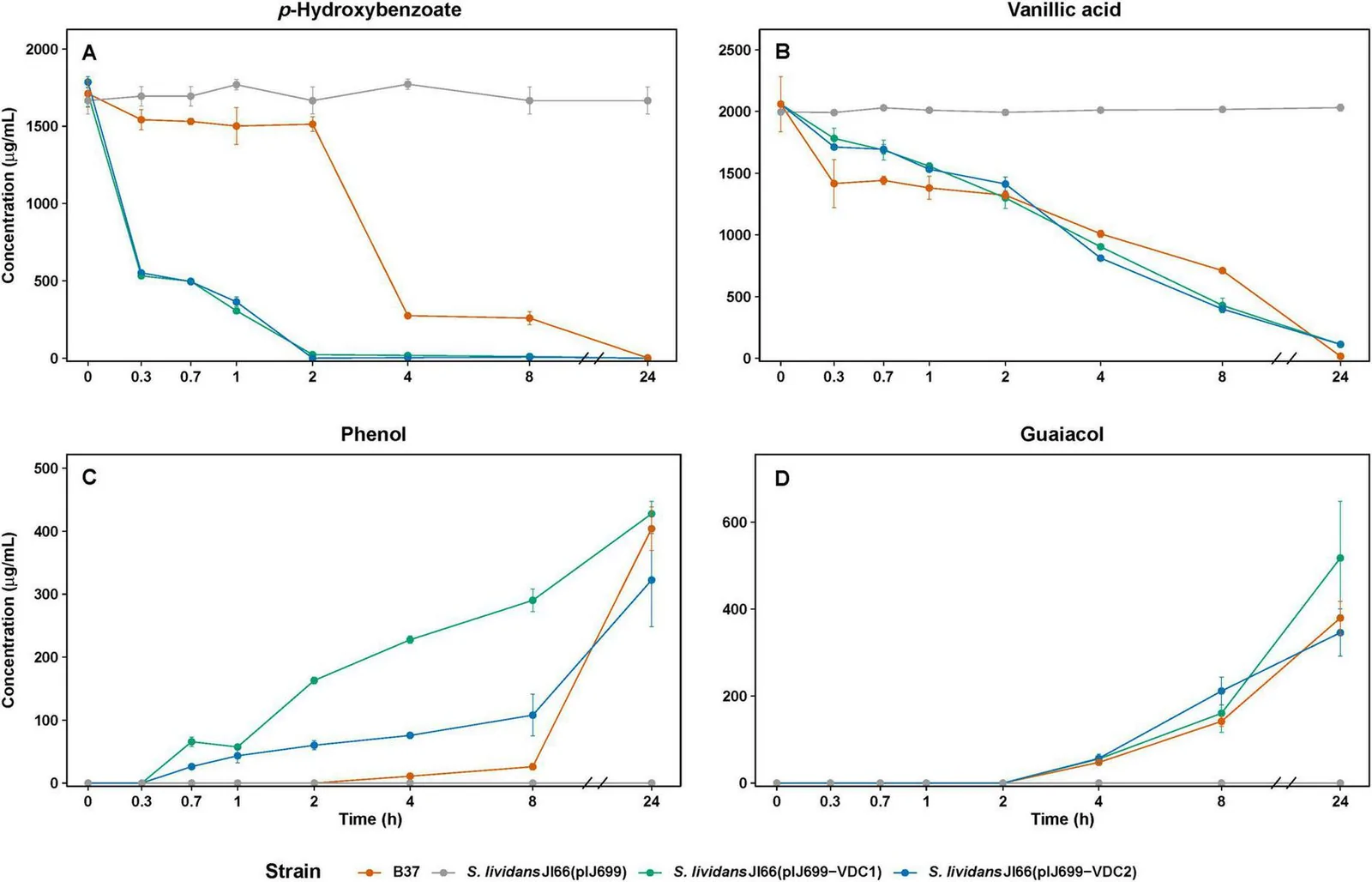
Functional validation of the *vdcBCD* cluster by aromatic acid decarboxylation assays. Time-course resting-cell biotransformation of *p*-hydroxybenzoate **(A)** and vanillic acid **(B)**, and corresponding production of phenol **(C)** and guaiacol **(D)** by strain B37, two independent *S. lividans* JI66 transformants carrying the B37 *vdcBCD* cluster, *S. lividans* JI66(pIJ699-VDC1) and *S. lividans* JI66(pIJ699-VDC2), and the empty-vector control *S. lividans* JI66(pIJ699). Compounds were quantified by HPLC using commercial standards. Data are means from three independent biological replicates after averaging technical replicates; error bars indicate standard deviation.

To experimentally address the role of the B37 VDC cluster in aromatic acid decarboxylation, resting-cell assays were performed using the parental strain B37 and two independent *S. lividans* transformants carrying the complete B37 *vdcBCD* gene cluster cloned in pIJ699, *S. lividans* JI66(pIJ699-VDC1) and *S. lividans* JI66(pIJ699-VDC2). A *S. lividans* transformant containing the empty pIJ699 vector, *S. lividans* JI66(pIJ699), was used as a negative control. All recombinant strains were assayed in the presence of 50 μg/mL thiostrepton to maintain plasmid selection. Substrate depletion and product formation were monitored over 24 h (Figure 7).

Linear mixed-effects models applied to log10(x + 1)-transformed concentrations revealed significant effects of strain, time, and their interaction for all four response variables (all *p* < 0.001; partial η^2^ ≥ 0.98), consistent with the strong strain-dependent differences observed throughout the resting-cell assays. Pairwise comparisons were based on estimated marginal means with Tukey or Sidak adjustment, as appropriate.

Non-inoculated substrate controls confirmed that *p*-hydroxybenzoate and vanillic acid remained stable under the assay conditions and did not give rise to detectable phenol or guaiacol, excluding abiotic substrate degradation or non-biological product formation (data not shown).

*S. lividans* JI66(pIJ699) maintained consistently high substrate concentrations throughout the assays and produced neither phenol nor guaiacol, confirming the absence of endogenous aromatic acid decarboxylation activity under the tested conditions in this species. In contrast, both *S. lividans* JI66(pIJ699-VDC1) and *S. lividans* JI66(pIJ699-VDC2) actively converted *p*-hydroxybenzoate and vanillic acid into phenol and guaiacol, respectively.

For *p*-hydroxybenzoate, both recombinant strains showed rapid substrate depletion (Figure 7A) and early phenol accumulation (Figure 7C), differing significantly from both B37 and *S. lividans* JI66(pIJ699) at the initial time points (Figure 7A). The parental strain B37 also biotransformed *p*-hydroxybenzoate, but displayed slower kinetics characterized by delayed substrate depletion (Figure 7A) and later phenol accumulation (Figure 7C). Nevertheless, by 24 h, *p*-hydroxybenzoate concentrations in B37 had decreased to levels comparable to those observed in both VDC transformants.

Phenol production mirrored these substrate-consumption patterns (Figure 7C). *S. lividans* JI66(pIJ699-VDC1) showed the strongest early phenol accumulation, whereas *S. lividans* JI66(pIJ699-VDC2) displayed a lower but clearly positive production profile. In contrast, B37 showed minimal phenol accumulation during the first hours of incubation followed by a marked increase at 24 h, reaching final phenol levels comparable to those observed in *S. lividans* JI66(pIJ699-VDC1).

Vanillic acid decarboxylation followed a slower and heterogeneous kinetic pattern than that observed for *p*-hydroxybenzoate (Figure 7B). B37 showed progressive vanillic acid depletion throughout the assay and displayed the most complete substrate depletion at the final time point. Both *S. lividans* JI66(pIJ699-VDC1) and *S. lividans* JI66(pIJ699-VDC2) also depleted vanillic acid relative to *S. lividans* JI66(pIJ699), particularly at intermediate and late time points, confirming that heterologous expression of the *vdcBCD* cluster conferred vanillic acid-transforming activity in *S. lividans*. However, depletion kinetics differed among strains, indicating that substrate conversion dynamics depend on the physiological and regulatory context in which the cluster is expressed.

Consistent with vanillic acid depletion (Figure 7B), guaiacol accumulated progressively in all active strains (Figure 7D) but remained undetectable in *S. lividans* JI66(pIJ699). Guaiacol production became evident from 4 h onward, with *S. lividans* JI66(pIJ699-VDC2) showing the highest accumulation at 8 h, whereas *S. lividans* JI66(pIJ699-VDC1) reached the highest final levels at 24 h. B37 displayed intermediate kinetics and reached final guaiacol concentrations comparable to those observed in *S. lividans* JI66(pIJ699-VDC2).

These results show that the *vdcBCD* cluster encodes a functional aromatic acid decarboxylation system active on both *p*-hydroxybenzoate and vanillic acid. Heterologous expression of the cluster in *S. lividans* JI66 conferred the ability to produce phenol and guaiacol from their corresponding aromatic acid substrates, whereas *S. lividans* JI66(pIJ699) showed no detectable substrate conversion. The quantitative differences observed between the two independent VDC transformants, particularly during vanillic acid transformation, further suggest that expression context, plasmid copy number, or transformant-specific physiological differences may influence the observed conversion efficiency.

Since whole resting-cell suspensions were used, substrate depletion and product accumulation should not be interpreted as a closed stoichiometric conversion. The assays were designed to demonstrate VDC-dependent formation of phenol and guaiacol, whereas the subsequent fate, recovery, or stability of these phenolic products under the assay conditions were not determined.

## 4 Discussion

This work directly extends previous observations from cork affected by yellow stain. In that system, cork alteration was associated with structural changes in cork tissues, distinct microbial colonization, and the detection of aromatic compounds related to lignin- and/or suberin-derived biodegradation, including *p*-hydroxybenzoate and phenol (Ruiz-Muñoz et al., 2025). Cork-derived *Streptomyces* isolates were also shown to transform *p*-hydroxybenzoate into phenol in resting-cell assays, suggesting a bacterial contribution to the formation or accumulation of phenolic intermediates in yellow-stained cork. The results presented in this work have identified the *vdcBCD* cluster as the genetic basis of that biotransformation by strain B37. Accordingly, the genomic and functional characterization of strain B37 was of particular interest to define the bacterial step responsible for phenol formation from *p*-hydroxybenzoate in yellow-stained cork. Genome sequencing and phylogenomic analysis supported the assignment of B37 to *S. graminifolii*, originally isolated from decaying vegetable material from a bamboo forest (Lee and Whang, 2014). Notably, B37 grouped with the *S. graminifolii* type-strain genome within a well-defined subclade. This group also included *S. colwelliae*, recently isolated as endophyte from root nodules of common alder (*Alnus glutinosa*) (Nouioui et al., 2025), *S. hokutonensis*, isolated from the rhizosphere of strawberry roots (Yamamura et al., 2014), and *S. prunicolor*, originally isolated from soil in Russia (Pridham et al., 1958).

Pangenome analysis indicated that B37 and the four closely related taxa included in this subclade carried genes encoding enzymes involved in aromatic compound metabolism that were absent or less represented in the genomes of other closely related *Streptomyces* species analyzed. Interestingly, all members of this subclade contained a complete *vdcBCD* gene cluster associated with non-oxidative aromatic acid decarboxylation. Thus, B37 belongs to a phylogenetically coherent lineage sharing the complete *vdcBCD* module and a broader accessory repertoire enriched in aromatic-compound-processing functions. Beyond the defining decarboxylation cluster, significantly enriched gene families and curated functional categories were mainly associated with hydroxybenzoate utilization, methoxylated aromatic metabolism, hydroxycinnamate-related pathways, ring-cleavage reactions, transport, regulation and redox-associated functions. By contrast, broad plant-polymer degradation categories did not dominate the comparative signal. We therefore refer to this phylogenetically coherent and functionally enriched lineage as the VDC^+^ genomic group.

Non-oxidative hydroxyarylic acid decarboxylation systems have been described in bacteria, including *Streptomyces* sp. (Chow et al., 1999), but few studies have linked these loci with phenotypes observed in environmental isolates. Here, we identified a conserved *vdcBCD* module in the cork-associated *S. graminifolii* B37 strain and showed that this cluster confers aromatic acid decarboxylation in *S. lividans*. Recombinant *S. lividans* strains carrying multiple copies of the *vdcBCD* cluster of strain B37 efficiently converted *p*-hydroxybenzoate into phenol and vanillic acid into guaiacol, whereas the empty-vector control showed no detectable conversion. To our knowledge, this extends the experimentally demonstrated substrate range of a *Streptomyces* VDC system, since the previously characterized *Streptomyces* sp. D7 *vdcBCD* cluster had been solely associated with vanillic acid decarboxylation into guaiacol (Chow et al., 1999).

However, this result was not entirely unexpected. Lupa et al. (2008) previously characterized a *Bacillus subtilis yclBCD* cluster encoding an enzyme complex capable of decarboxylating both vanillate and *p*-hydroxybenzoate under aerobic and anoxic conditions. Interestingly, the affinity of this enzyme complex was four-fold higher for *p*-hydroxybenzoate than for vanillic acid. In our resting-cell assays, *S. lividans* transformants carrying the *vdcBCD* cluster converted *p*-hydroxybenzoate more rapidly than vanillic acid, suggesting a similar behavior to that previously observed in *B. subtilis*. These results indicate that, under the tested conditions, the B37 VDC system transformed *p*-hydroxybenzoate more readily than vanillic acid.

The organization of the B37 *vdcBCD* locus is consistent with that previously described for bacterial non-oxidative hydroxyarylic acid decarboxylase/phenol carboxylase systems. These systems are typically encoded by three clustered genes, *vdcB*, *vdcC* and *vdcD*, with *vdcB* encoding a UbiX-like flavin prenyltransferase, *vdcC* encoding a UbiD-like decarboxylase, and *vdcD* encoding a small accessory component (Lupa et al., 2005, 2008). The sizes and annotations of the B37 proteins closely matched this canonical organization. VdcB and VdcC were highly conserved across the VDC^+^ genomes, whereas VdcD showed greater sequence divergence. The retention of *vdcD* in all genomes carrying the complete module is relevant because this component is required for activity in characterized BCD systems despite its lower sequence conservation. In *Streptomyces* sp. D7, deletion of *vdcD* abolished vanillate decarboxylation (Chow et al., 1999), and structural work on vanillic acid decarboxylase later demonstrated that VdcD acts as an allosteric activator of the UbiD-like VdcC subunit (Marshall et al., 2021). The conservation of the complete three-gene architecture across the VDC^+^ genomes therefore supports its interpretation as a functional aromatic acid decarboxylation module.

The kinetic differences between the recombinant *S. lividans* transformants and native B37 are likely due to the higher gene dose of the *vdcBCD* cluster in the transformants (several copies versus one single copy in B37). However, differences in expression context are also likely to contribute. The VDC transformants rapidly depleted *p*-hydroxybenzoate and accumulated phenol, whereas B37 showed delayed substrate depletion and later product accumulation. This pattern is compatible with the heterologous expression context, since expression from a multicopy plasmid in *S. lividans* may differ from the native regulatory context of the cluster in B37. The association of the *vdcBCD* locus with AraC- and MarR-family regulators, transport-related genes and oxidoreductases further suggests that this module is embedded in a local genomic context related to aromatic compound sensing, uptake and transformation. Comparable regulatory control has been described for aromatic catabolic pathways in *Streptomyces*, including the β-ketoadipate pathway in *S. coelicolor*, where pathway-specific regulators control genes required for protocatechuate catabolism (Davis et al., 2013). *p*-Hydroxybenzoate catabolism in *S. coelicolor* is also regulated by dedicated transcriptional control, with *p*-hydroxybenzoate being converted into protocatechuate before entering central aromatic catabolism (Zhang et al., 2018). In B37, the *vdcBCD* locus may therefore represent an alternative or complementary route for aromatic acid transformation, based on non-oxidative decarboxylation rather than hydroxylation followed by ring-cleavage metabolism.

**FIGURE 8:**
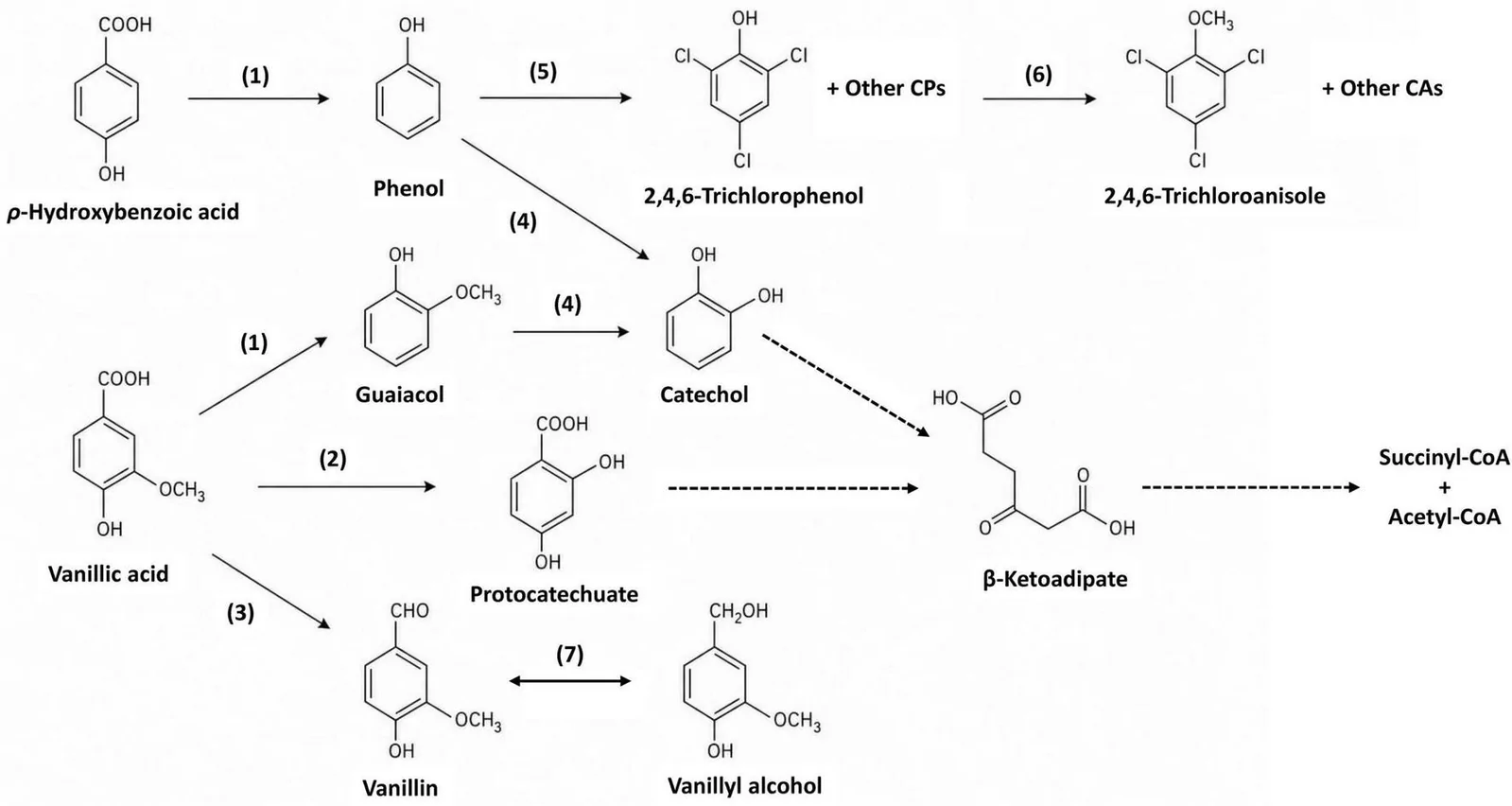
Proposed microbial biotransformation and degradation network linking *p*-hydroxybenzoate and vanillic acid with phenolic derivatives, chlorinated compounds and the β-ketoadipate pathway. Solid arrows indicate the main biotransformation reactions, while dashed arrows represent downstream ring-cleavage steps leading to β-ketoadipate and central metabolism. Numbered enzymes are: vanillate/*p*-hydroxybenzoate decarboxylase (1), vanillate *O*-demethylase (2), carboxylic acid reductase (3), phenol hydroxylase/guaiacol *O*-demethylase (4), chloroperoxidase (5), chlorophenol *O*-methyltransferase (6), and vanillin oxidoreductase (7).

Aromatic acid transformation in actinomycetes can proceed through alternative routes depending on the enzymatic repertoire and regulatory context of each strain. In *Streptomyces* sp. D7, vanillate is converted to guaiacol through the *vdcBCD* system (Chow et al., 1999). A comparable role for *vdcBCD* has been shown in *Amycolatopsis* sp. ATCC 39116, where deletion of the *vdcBCD* cluster abolished guaiacol formation and strongly impaired growth on vanillic acid, whereas deletion of the vanillate demethylase cluster *vanAB* had only a minor effect (Figure 8; Meyer et al., 2017). This suggests that non-oxidative decarboxylation can constitute an important vanillate-transformation route in some actinomycetes. At the same time, protocatechuate-linked pathways are also present in *Streptomyces* and can support downstream processing of hydroxybenzoate-derived aromatic intermediates (Figure 8; Iwagami et al., 2000; Davis et al., 2013; Zhang et al., 2018). The dual activity of the B37 VDC complex, acting on vanillic acid and *p*-hydroxybenzoate to produce guaiacol and phenol, respectively, places VDC complex cluster as a branching point in aromatic acid transformation (Figure 8). Once formed, phenol can undergo degradation by being transformed into catechol in a reaction catalyzed by the enzyme phenol hydroxylase. Consistent with this possibility, the B37 genome contains a putative FAD-binding monooxygenase of the PheA/TfdB family, that could be involved in catechol formation, although this aspect remains to be further investigated. Phenol hydroxylases are inducible, NADPH-dependent flavoprotein monooxygenases that catalyze the conversion of toxic phenol into catechol, enabling actinomycetes such as *Streptomyces setonii* to decompose industrial pollutants (Antai and Crawford, 1983).

However, part of the phenol may be released from the cell, as suggested by its detection in appreciable quantities in the resting-cell experiments performed, thereby escaping degradation through catechol formation. In this case, phenol can be secondarily used as a substrate by chloroperoxidases to generate CPs, including 2,4,6-TCP (Figure 8; Ruiz-Muñoz et al., 2025). In fact, the genome of B37 strain contains several genes encoding chloroperoxidase-like enzymes (Figure 5). This product is the direct precursor of 2,4,6-TCA, which is formed by *O*-methylation of 2,4,6-TCP in a reaction catalyzed by fungal chlorophenol-*O*-methyltransferase (Coque et al., 2003; Feltrer et al., 2010). Therefore, phenol metabolism could be channeled through a degradative pathway, via conversion into catechol, or, more nonspecifically, toward the formation of a variety of CPs which, due to their greater intrinsic toxicity, would be detoxified through transformation into CAs.

Thus, the B37 VDC system is most consistent with downstream processing of soluble aromatic compounds in chemically altered cork. Lignin and suberin modification can generate heterogeneous mixtures of low-molecular-weight aromatic compounds, and bacteria may participate in the transformation or assimilation of these soluble intermediates after extracellular release. This interpretation is consistent with studies showing that some *Streptomyces* strains can modify lignocellulosic material, or grow on lignin-derived substrates, while also carrying intracellular pathways for aromatic compound metabolism (Crawford et al., 1983; Pometto and Crawford, 1986; Riyadi et al., 2020; Tan et al., 2022). In the case of B37, the validated reaction contributes specifically to the conversion of aromatic acids into phenolic products. This activity may diversify the pool of cork-derived aromatic intermediates and could operate alongside additional enzymatic activities, growth conditions or members of the cork microbial community that further transform these compounds.

This peripheral-transformation role is compatible with the biological-funneling model, in which diverse plant-derived aromatics undergo initial peripheral reactions before entering central ring-cleavage pathways (Linger et al., 2014). Within this framework, VDC-like decarboxylation represents a branching reaction that can divert aromatic acids toward phenolic products rather than directly toward protocatechuate- or catechol-linked ring cleavage. In cork-associated environments, such reactions could contribute to the accumulation of phenol and methoxyphenols derived from lignin- and/or suberin-associated aromatic precursors. This is particularly relevant for yellow-stained cork, where *p*-hydroxybenzoate and phenol were previously detected and where a multi-organism route was proposed for the formation of CPs and CAs from plant-derived aromatic precursors (Ruiz-Muñoz et al., 2025). The present study identifies a genetically defined and experimentally validated bacterial step within that proposed route. Because phenol can act as a precursor in the formation of CPs and CAs, *p*-hydroxybenzoate decarboxylation by B37 and related strains may represent a relevant bacterial step within the chemical sequence proposed for yellow-stained cork and cork-taint-related compounds.

Complete *vdcBCD* clusters should also be distinguished from isolated UbiX- or UbiD-like homologues. Homologues of the B and C components of these systems are broadly distributed across bacteria, archaea and eukaryotes, whereas homologues of the D component and complete BCD gene clusters are less frequent (Lupa et al., 2005). Therefore, individual UbiX- or UbiD-like homologues should not be considered equivalent to a complete functional *vdcBCD* module. Within the comparative framework analyzed here, the complete *vdcBCD* cluster was conserved in B37 and its closest genomic relatives, supporting the idea that this group shares a complete aromatic acid decarboxylation module. Broader sampling across *Streptomyces* will be required to determine whether this distribution reflects vertical inheritance, ancient acquisition followed by differential retention, horizontal transfer, or a combination of these processes.

This study identifies VDC-dependent aromatic acid decarboxylation as a key bacterial step in the transformation of cork-derived aromatic acids by *S. graminifolii* B37. The activity was validated by heterologous expression and occurs within a conserved genomic background shared by closely related VDC^+^ strains. These findings support a role for B37 and its closest relatives in the transformation of soluble aromatic acids in altered cork, rather than in primary cork-polymer degradation. Whether this pathway is expressed in the cork matrix, and how much it contributes to phenol accumulation during yellow-stain development, remains to be tested. Experiments with cork-relevant substrates, expression analyses and targeted mutagenesis in B37 are now needed to determine how this decarboxylation step integrates with lignin and suberin modification, phenol halogenation, and *O*-methylation during cork alteration in YS.

## Data Availability

The complete genome sequence of *Streptomyces graminifolii* B37 (deposited in NCBI as *Streptomyces* sp. YS-B37) is available under GenBank accession number CP184648.1. The 11 *Streptomyces* type strain genomes used for comparative pangenome analysis were downloaded from NCBI; their accession numbers and the selection criteria are provided in Supplementary Table 1. All R scripts used for analysis, statistical modeling, and figure generation are available at GitHub: https://github.com/marinaruizmunoz/B37-vdcBCD-manuscript-analyses.

## Footnotes

1
https://tygs.dsmz.de

2
https://cagecat.bioinformatics.nl/tools/clinker
